# Embedding AI-Enabled Data Infrastructures for Sustainability in Agri-Food: Soft-Fruit and Brewery Use Case Perspectives

**DOI:** 10.3390/s24227327

**Published:** 2024-11-16

**Authors:** Milan Markovic, Andy Li, Tewodros Alemu Ayall, Nicholas J. Watson, Alexander L. Bowler, Mel Woods, Peter Edwards, Rachael Ramsey, Matthew Beddows, Matthias Kuhnert, Georgios Leontidis

**Affiliations:** 1 Interdisciplinary Institute, University of Aberdeen, Aberdeen AB24 3FX, UK; georgios.leontidis@abdn.ac.uk; 2 School of Natural and Computing Sciences, University of Aberdeen, Aberdeen AB24 3UE, UK; a.li.21@abdn.ac.uk (A.L.); tewodrosalemu.ayall@abdn.ac.uk (T.A.A.); p.edwards@abdn.ac.uk (P.E.); m.beddows.21@abdn.ac.uk (M.B.); 3 School of Food Science and Nutrition, University of Leeds, Leeds LS2 9JT, UK; n.j.watson@leeds.ac.uk (N.J.W.); a.l.bowler@leeds.ac.uk (A.L.B.); 4 Duncan of Jordanstone College of Art and Design, University of Dundee, Dundee DD1 4HT, UK; m.j.woods@dundee.ac.uk; 5 SAC Consulting, Scotland’s Rural College, Edinburgh EH9 3JG, UK; rachael.ramsey@sac.co.uk; 6 Institute of Biological and Environmental Sciences, University of Aberdeen, Aberdeen AB24 3UU, UK; matthias.kuhnert@abdn.ac.uk

**Keywords:** artificial intelligence, Internet of Things, net zero, agri-food

## Abstract

The agri-food sector is undergoing a comprehensive transformation as it transitions towards net zero. To achieve this, fundamental changes and innovations are required, including changes in how food is produced and delivered to customers, new technologies, data and physical infrastructures, and algorithmic advancements. In this paper, we explore the opportunities and challenges of deploying AI-based data infrastructures for sustainability in the agri-food sector by focusing on two case studies: soft-fruit production and brewery operations. We investigate the potential benefits of incorporating Internet of Things (IoT) sensors and AI technologies for improving the use of resources, reducing carbon footprints, and enhancing decision-making. We identify user engagement with new technologies as a key challenge, together with issues in data quality arising from environmental volatility, difficulties in generalising models, including those designed for carbon calculators, and socio-technical barriers to adoption. We highlight and advocate for user engagement, more granular availability of sensor, production, and emissions data, and more transparent carbon footprint calculations. Our proposed future directions include semantic data integration to enhance interoperability, the generation of synthetic data to overcome the lack of real-world farm data, and multi-objective optimisation systems to model the competing interests between yield and sustainability goals. In general, we argue that AI is not a silver bullet for net zero challenges in the agri-food industry, but at the same time, AI solutions, when appropriately designed and deployed, can be a useful tool when operating in synergy with other approaches.

## 1. Introduction

The global food system contributes about one third of anthropogenic greenhouse gas (GHG) emissions [1] and must be part of a rapid transition towards net zero. Food and drink is the largest and most socially significant manufacturing sector in the UK, with approximately 11,000 businesses employing 430,000 people and contributing GBP 35bn to the national Gross Value Added (GVA) each year [2], with the entire agri-food sector accounting for a total estimated GBP 128.3bn [3]. The sector’s contribution to climate change is not homogenous for every product, or even for the same product produced in different locations or by different producers. Delivery against ambitious net zero targets will require a range of measures to be adopted across the agri-food supply chain.

Over the last few decades, rapid advances in processes to collect, monitor, disclose, and disseminate information have contributed towards the development of entirely new modes of environmental governance for supply chains. Transparency helps different supply chain actors identify and minimise risks and determine whether and where progress is being made. GHG emissions are usually quantified through modelling or measurements (direct and indirect). There are a variety of options to quantify GHG emissions for different production processes, including sustainability scorecards [4], self-disclosure systems [5], process-based models [6,7], and various empirical/process-based carbon calculator tools [8,9]. However, such instruments may suffer from low accuracy or high uncertainty, require expert knowledge, or are data-intensive and laborious to use.

Two commonly used approaches for reducing the carbon footprint of products are either to produce more with the same resources and dimensions or to make the current production more sustainable and efficient, reducing the quantity of inputs associated with a high carbon footprint (e.g., fertilisers). However, such approaches are not mutually exclusive and it is plausible to assume that current food manufacturing processes can still be optimised so the production (e.g., crop yields) is maximised with minimal inputs, and the degradation of current resources is reduced. Artificial Intelligence (AI) and Internet of Things (IoT) are often considered technologies that could potentially assist with such tasks; however, more research is needed to fully understand their potential [10].

In this article, we reflect on some of our experiences with user-centred AI-based solutions for reducing the impact of carbon emissions on food producers, with an emphasis on transparency and real-world testing in the UK context. We focus on two main questions: *Can sensors and AI technologies be used by food and drink producers in the agri-food sector to lower their emissions? What are the barriers preventing the adoption of sensors and AI technologies by the end users in this context?* We investigate these questions in the context of two distinct agri-food use cases involving strawberry and beer production that each present sets of common and unique challenges, reflecting the highly heterogeneous nature of the UK agri-food sector. Our experience demonstrates that future AI-based solutions will have to be embedded in complex socio-technical contexts that present significant challenges, such as data quality, the scaleable monitoring of production processes at a required level of detail, domain shifts linked to changes in grown (e.g., new plant varieties) and manufactured products (e.g., a new line of beers), unexpected events (e.g., changes in weather patterns due to changing climate), and the reusability of models across different farms and production facilities. Our main contribution is a joint interdisciplinary perspective on significant challenges preventing impactful AI deployment in agri-food for non-typical application use cases grounded in recent experience with real-world stakeholders. Given the heterogeneity of the agri-food sector in the UK and the ambitious net zero deadlines, we believe that such interdisciplinary perspectives set an important agenda for future AI solutions.

## 2. Motivating Use Cases

The food and drink manufacturing sector is remarkably diverse in its production processes, with over 30 distinct subsectors and approximately 97% of businesses being small and medium-sized enterprises (SMEs). While the focus of sustainability research is often aimed at staple foods, non-essential but high-value products, for example, strawberries and beer, are not always considered. Below, we describe two use cases that we have explored to evaluate the potential of new sustainability data insights and actions delivered through a combination of IoT sensing and AI. In these use cases, we have studied how AI can be applied at different stages of the food supply chain. For example, in the strawberry use case, we focus on farm-to-gate production, while in the beer use case, we focus on the brewing processes taking place in the brewery.

### 2.1. Strawberry Production

**Figure 1:**
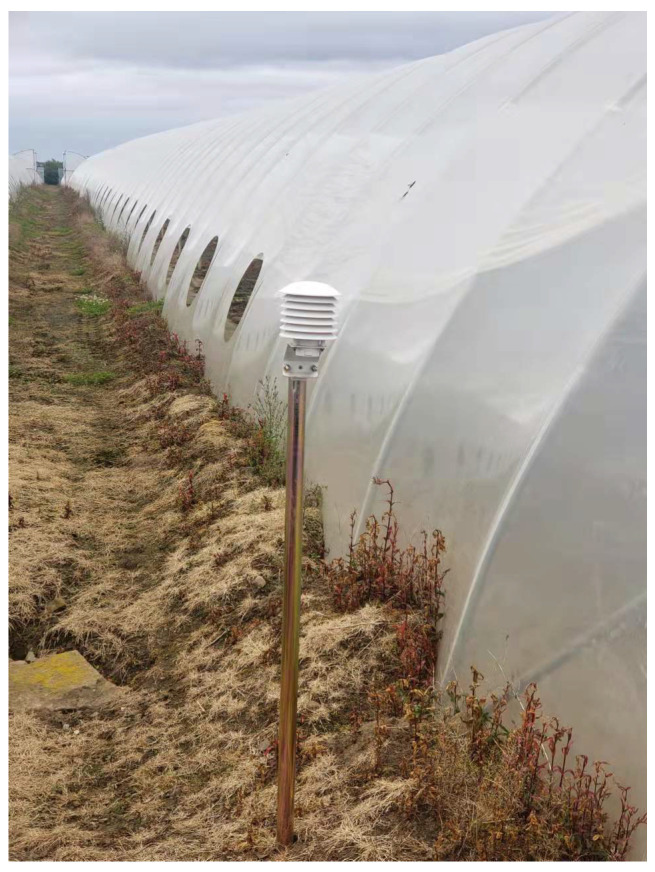
Temp./humidity sensor outside tunnel.

**Figure 2:**
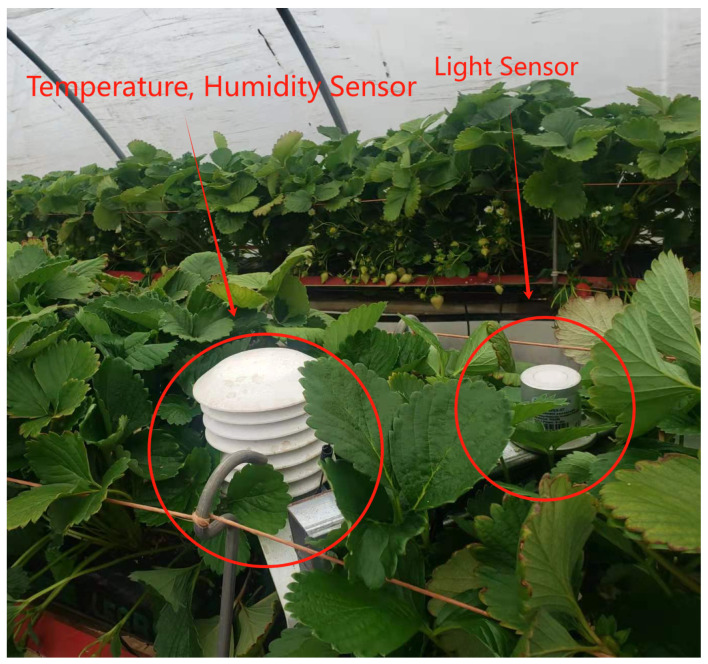
Temp./humidity and light sensor inside tunnel.

**Figure 3:**
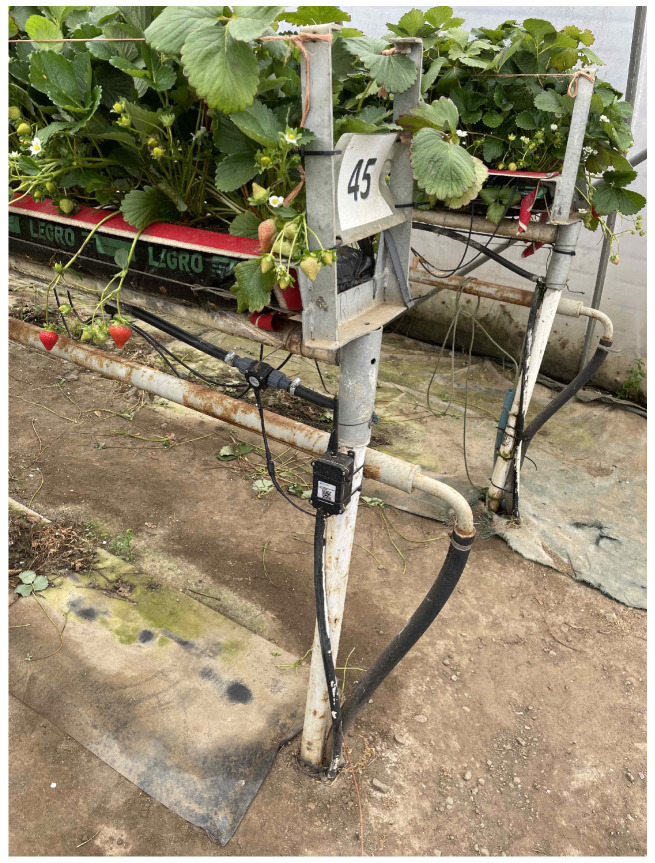
Flow meter inside tunnel.

We partnered with a major UK strawberry producer to develop and evaluate real-world deployments of IoT-enabled AI sustainability platforms which aim to maximise strawberry production while maintaining the lowest possible carbon footprint. One of the typical setups for strawberry production in Scotland is a large polytunnel with 4–5 rows of growing tables. The strawberry plants are grown in bags containing coir, and nutrients are delivered through a connected irrigation system. In our experiments, we deployed a number of IoT sensors to measure the environmental conditions inside and outside of the polytunnel. These included:

- *Outside Temperature/Humidity Sensors* (R711-Wireless Temperature and Humidity Sensors are used to monitor both indoor and outdoor temperature and humidity): Positioned outside each tunnel (Figure 1) to monitor the external temperature (°C) and humidity (%). Data are recorded at 20 min intervals.
- *Inside Temperature/Humidity Sensors*: Located within the tunnels (Figure 2) to record the internal temperature (°C) and humidity (%). Data from this sensor are recorded at 20 min intervals.
- *Light Sensors* (a Dragino WSC1-L Weather Station is deployed to record light): Used to measure the amount of photosynthetically active radiation (PAR) the plants are exposed to throughout the day at 20 min intervals, with PAR measured in units of μmol/m^2^s. These are located inside the polytunnels (Figure 2) and are powered by batteries which are re-charged by solar panels.
- *Soil Condition Sensors* (a Dragino Soil Moisture Sensor LSE01 is deployed for soil measurements): These measure specific soil parameters including the temperature (°C), moisture (%), and conductivity of the soil. The sensor probe is inserted 10cm into the soil and the data are updated at 20 min intervals.
- *Flow Meter* (a Dragino SW3L Outdoor Flow Sensor Smart Rural is used for measuring the water flow): This sensor (Figure 3) records the volume of water, measured in litres (L), used for irrigating the plants. Data are updated at 20 min intervals, providing detailed usage patterns throughout the day.

The data provided by such sensors are useful for monitoring the growth cycle of plants and, in combination with additional information (e.g., historical yield data), can be used to train prediction models for yield forecasting [11]. Additionally, in this use case, the values reported by the water flow meters can also be used as a proxy measurement for the amount of fertiliser used in production (i.e., irrigation rigs tend to pump a specific mix of water and fertiliser during the season) and the electricity spent by the irrigation pumps.

From the carbon footprint perspective, there are a number of potential uses of such sensor data. For example, light sensor readings can be used to discover changing properties of ageing polytunnel plastic. A potential reduction in carbon footprint would result from delaying the replacement of the plastic beyond its original estimated lifespan based on empirical measurements rather than pre-set values. However, the deployment of static sensors in all tunnels solely for this reason would be undesired, as the sensors themselves carry a carbon footprint which could offset the potential savings. Another portion of the carbon footprint of strawberry farming is generated through the energy consumption of the irrigation rig and the use of fertilisers in the irrigation solution. A yield-forecasting model capable of predicting optimal water use for maximum yield would potentially reduce the amount of irrigation and thus reduce the use of energy and fertilisers.

### 2.2. Brewery

**Figure 4:**
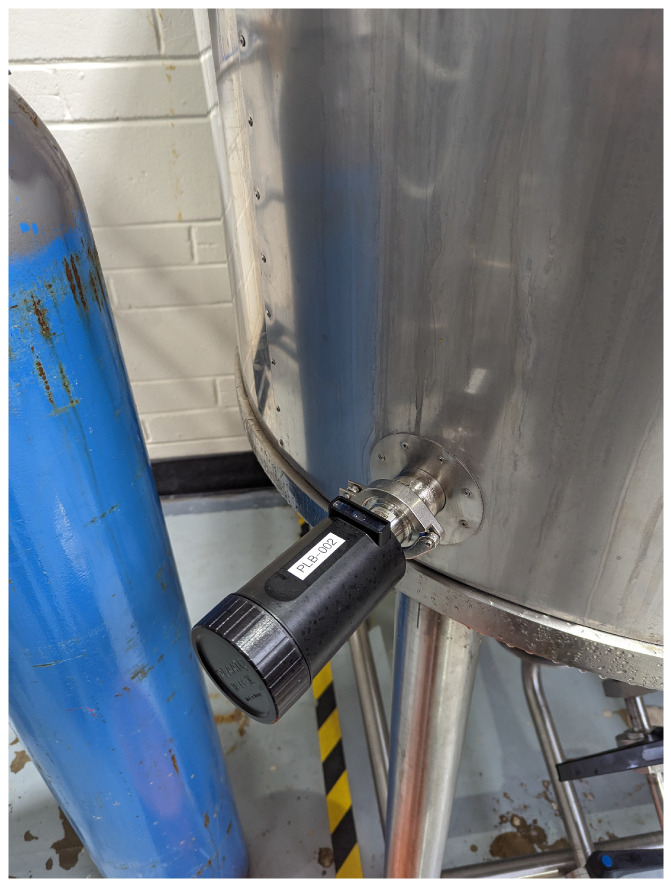
Fermentation sensor.

**Figure 5:**
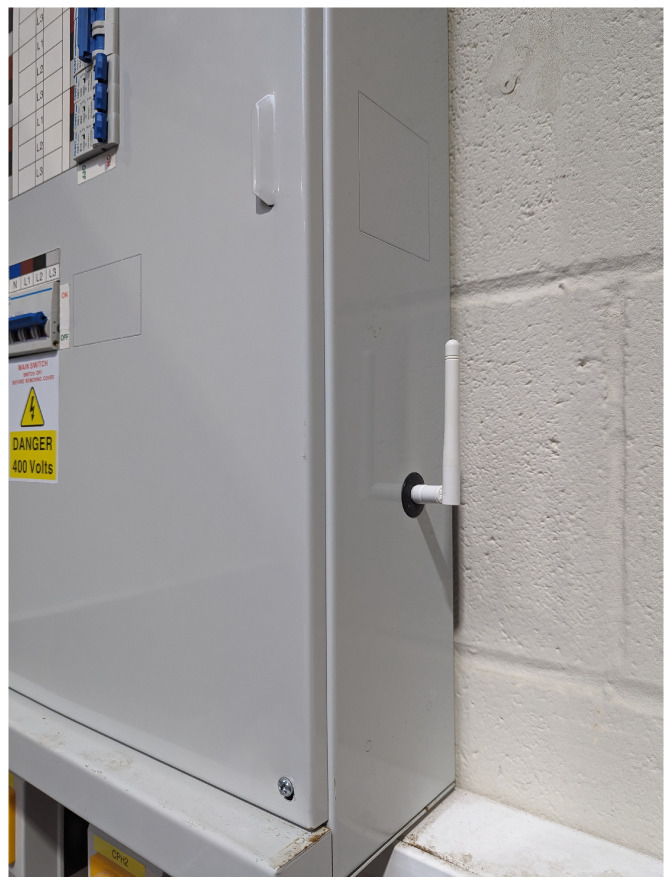
Wireless electricity monitor.

We partnered with a craft brewery located in Leeds, UK, to show how the implementation of low-cost sensing solutions combined with AI can improve the quality of the brewing process while reducing the carbon footprint. The brewing process starts with mashing, where malted grains are soaked in hot water to activate enzymes that convert starches into fermentable sugars. After mashing, this mixture, known as the wort, is boiled with hops for flavouring, then cooled and fermented with yeast to produce alcohol. Fermentation is the most important step in the beer brewing process because this is where the yeast consumes the fermentable sugars derived from malted grains and produces alcohol and carbon dioxide as byproducts. This not only gives the beer its alcohol content, but also contributes significantly to its flavour, aroma, and carbonation. The sensors we installed included the following:

- *Fermentation sensors*: These were tuning-fork technology sensors that measure the density (SG) and temperature (°C) of the fermenting wort (Figure 4). Readings are taken every 30 min.
- *Electricity monitor*: This measures the electricity consumption (kW) of each stage of the brewing process (Figure 5). Data are recorded at 1-s intervals.
- *Water meter*: This measures the total water consumption (m^3^) of the brewing process. Data are recorded for every cubic meter of water consumed.

The data provided by the sensors are useful to monitor the fermentation process and to achieve a better and more consistent product quality. Real-time sensor data (e.g., electricity and water consumption) can also serve as inputs into calculators to determine the carbon footprint of the brewing process (e.g., per batch). More sophisticated AI models can be trained using the fermentation sensor data to predict the fermentation end time. This can enable more efficient equipment scheduling and lower energy usage by reducing the time required for the fermented product to be cooled [12]. AI models combining electricity and water energy consumption data with historic production schedules can be used to predict the electricity and water usage at each stage of the brewing process. This information can then be used to input into the carbon footprint calculator, identify process hotspots, and set baselines for anomaly detection.

## 3. Role of User Engagement and Creative Thinking

User engagement plays a vital role in ensuring products and services meet the needs of stakeholders. Often, scientific solutions target an optimum quantification of GHG emissions but do not necessarily consider the practical applicability. Taking available data and human behaviours into account from the beginning represents real-life applications rather than serving as a scientific tool. An example of this is GHG emission calculators that are developed based on the available data, and are typically empirical-based models, in contrast to more academic-orientated process-based models. Process-based models, while potentially more accurate, cannot be applied without additional specific measurements on the farm, which farmers do not routinely collect.

Design thinking is a process that can be defined through five key steps: empathise, define, ideate, prototype, and test [13]. Understanding user needs is the foundation for gathering robust user requirements. Promoting user engagement from the outset can support early insights into the preferences, challenges, and opportunities for innovation. Continuing to involve stakeholders, using a co-design approach, can support development through iterative feedback, the validation of concepts, and the testing of prototypes. This approach reduces risk and enhances the uptake. In our project, we organised workshops and sessions that included soft-fruit growers and brewery stakeholders who iteratively mapped their supply chains to identify opportunities and gaps for AI technology solutions. Participants also explored future scenarios and their motivations regarding pro-environmental sustainability. Their continuous input was paramount in ascertaining that all our developments and outputs are useful to them, and therefore easier to adopt in real life. Specifically, understanding stakeholder requirements helped us to understand the data gaps within the use case scenarios which could be addressed through sensor deployments. As a result, an external company was sourced to deploy and maintain the sensors throughout two seasons for the strawberry use case. For the brewery use case, the sensors were deployed internally by the breweries and monitored multiple production batches. In addition to the sensor data, stakeholders provided additional information detailing their processes in the form of spreadsheets (e.g., yield data) and manual reports.

Discussions with end users (e.g., farmers), service providers (e.g., developers of carbon calculators), and policy makers during workshops and stakeholder visits have enabled us to uncover many of the social and economic barriers discussed in this paper. More technical challenges were discovered, mostly through our ongoing work, on prototype software infrastructures. This is an important step in a user-centred development process as it offers tangible outcomes that enable the end users to better understand and evaluate candidate solutions to previously identified challenges. Our software prototypes included a sensor-assisted yield-forecasting AI tool; a carbon footprint calculator aimed at craft breweries [14]; and a provenance toolkit for enhancing the transparency of carbon emission calculations using semantic technologies [15,16].

## 4. Carbon Footprint Calculations

Sustainability efforts are dependent on accurate and consistent monitoring of environmental impacts. Today, various business activities and products can be associated with quantitative estimates of their impact in terms of GHG emissions. For example, the footprint of a food product includes the scope emissions of the entire supply chain, which includes the upstream production of required goods (e.g., seeds, fertiliser), production of the food, processing of the product, packaging, waste, and all transport involved. It is therefore an aggregation of emissions produced by individual business activities that influence the products. While some of these emission footprints can be measured directly, some need to be modelled or estimated. However, it is not always easy to assess what methods and data were used to derive such estimates.

### 4.1. Transparency of Carbon Footprint Calculations

A significant portion of Net Zero targets are evaluated against quantifiable goals, typically expressed in CO_2_-equivalent (CO_2_e). Measurements of emissions are complex, costly, and difficult to apply. Therefore, several methods have been developed to calculate emissions (e.g., modelling or empirical analysis). These life cycle analysis emission calculation methods that were previously used mostly in the context of academic research, with understood and defined limitations, are now being embedded within commercial solutions such as carbon footprint calculators, which often rely on manual user input (e.g., estimate the amount of electricity used). The estimated CO_2_e emissions associated with agri-food activities are increasingly becoming linked to the constraints and financial burden of the sector and may be used as a basis for future policy interventions and quotas that may impact the future capacity of the food supply chain. In such a context, organisations may have a vested interest in deliberately under-reporting certain aspects of their carbon footprint if they deem it could have a negative impact on the company image [17]. Lynch et al. [18] also argue that emissions produced by agriculture are different to those produced by industries heavily dependent on fossil fuels, and hence, current reporting methods using CO_2_e values are insufficient. With non-CO_2_ gases such as methane and nitrous oxide forming a significant portion of emissions, different and more precise calculation methodologies, or indeed, metrics, might be needed in the future. However, without transparent and detailed data provenance records, it will be challenging to understand how the emission calculation methodologies are applied to produce carbon footprint estimates.

To address these challenges, the implementation of sensors across stages of the agri-food supply chain becomes critical. Sensors potentially offer more accurate input data and information in comparison to various estimates which are being used currently. Such estimates are often calculated indirectly using assumptions such as when the whole farm energy use is allocated to products/enterprises based on averages. On the other hand, sensors such as energy monitors can directly track electricity consumption, and water meters can accurately measure water use, removing the potential to under- or over-report these figures. Using sensors ensures that the data collected are reflective of actual consumption, but also secures a reliable dataset for future reference should emission calculation methodologies change. The raw sensor data may then be fed into carbon footprint calculators, which employ additional calculation methods (e.g., emission conversion factor multiplications) to produce carbon footprint estimates.

However, while monitoring energy and water consumption is accurate and the associated conversion factors are robust, other emissions are more difficult to measure. For example, direct nitrous oxide (N_2_O) flux measurements are very spatially and temporally variable, requiring either intensive labour or expensive equipment for direct measurement, and are beyond the scope of a typical sensor on a farm. However, because of the high global warming potential, these fluxes greatly impact overall emissions. Considering the potentially large error in the measurements, indirect factors that estimate fluxes based on the applied amount of nitrogen will provide comparable results. It is therefore crucial to understand the various assumptions and parameters of such calculation methods, their interdependencies, and the real-life impact of bias and the uncertainty of the produced CO_2_e estimates. Such an improved understanding of these implications would allow the promotion of more accurate estimates of the achieved emission reductions, and what remains to be done to achieve net zero.

### 4.2. Granularity of GHG Calculations

GHG calculations can be calculated to different methodological guidelines and frameworks for different spatial and temporal scales of application, i.e., the country inventory level, sectoral level, farm carbon footprint level, enterprise level, and product level). The target scale depends on the objective. While a user might be interested in the carbon footprint of a box of strawberries, the more relevant question for farmers may be the emissions on the farm over a one-year cycle. The differentiation of the carbon footprint for the different sections or scales is not always possible with the available data and methods. However, AI tools that aim to support decision-making for optimising environmental impacts against business objectives may require an ability to quantify the carbon footprint at a highly granular level. For example, a machine learning model that predicts the yield for individual polytunnels needs to be able to quantify the carbon footprint on a per-tunnel basis. For this, records quantifying energy consumption, water, and fertiliser use for an individual tunnel need to exist.

In the brewing case study, real-time fermentation, electricity monitors, and water meters allow for accurate carbon footprint quantification per brew. Furthermore, the estimations of the energy use of each brewing stage supported by the electricity monitors recording energy use for individual heating elements (e.g., boiler and hot liquor tank), along with AI methods combined with historical production schedules, allow for improved estimation of the contribution of each brewing stage to the overall carbon footprint. This stage-specific quantification of emission footprint contributions allows the detection of hot-spots (strongest contributors) and is necessary for efficient optimisation strategies.

### 4.3. Open-Source Carbon Footprint Accounting

Many businesses find it challenging to evaluate the carbon footprint across their entire value chain, a crucial step towards identifying and implementing the most effective sustainability practices. Transparent, open-source, freely available carbon footprint calculators can enable producers to compare mitigation options across scope 1, 2, and 3 emissions as opposed to only minimising more immediate options (e.g., reducing energy consumption or fertiliser use). An example of this is our open-source carbon footprint calculator aimed at breweries [14]. These calculators can demystify the process of carbon footprinting and enable managers to understand their limitations and uncertainties. These calculators close the knowledge gap between business managers and sustainability consultants, empowering managers to make informed decisions themselves that not only enhance the sustainability of their operations but also bring about economic benefits (e.g., reduced energy and water consumption, minimised packaging use, and the adoption of renewable energy sources). Facilitating the implementation of solutions that offer economic benefits alongside environmental improvements encourages more rapid adoption of sustainable practices and fosters a culture of innovation and sustainability within these industries. Furthermore, tool transparency allows users to engage with any changes in calculation methods (e.g., emission factors), enabling direct comparison between businesses now and in the future.

## 5. Environment Volatility and Need for Resilient Solutions

The use of AI in agri-food is linked to a range of data-related challenges [19]. These challenges are not merely technical, but also involve aligning with the needs and interests of all stakeholders, including farmers. The data are critically dependent on farmers’ participation, which is often influenced by perceived benefits and the practicalities of their daily operations. Additionally, deploying sensors and technologies on a large scale imposes a substantial capital cost, and yet, sometimes, even current sensing technologies may struggle with precision and reliability.

Transitioning from data collection and technological deployment, we often encounter further challenges in the application of machine learning in agri-food. Based on our own experience, traditional machine learning models, while powerful in analysing and predicting based on historical data, often struggle with the unpredictability of incidents in agri-food, such as heatwaves, supply chain issues, and diseases. For instance, a yield prediction model may show excellent performance in a controlled environment; however, it can struggle to account for external disruptions like a sudden outbreak of mildew that reduces yield. This is because the model is trained on historical environmental data such as weather and may lack the features to account for the impact of diseases. Moreover, the transferability and generalisability of a model is to be considered. The performance may dip and struggle to adapt when applied to a different setting, such as another polytunnel or another farm with a different configuration or crop variety. This is in contrast with the brewery use case where processes tend to remain similar across the different organisations.

Data heterogeneity can be also a driving factor in disrupting the generalisation of models, particularly due to environmental conditions and agricultural practices. For example, variations in soil types and climate between farms can drastically alter crop growth conditions, making a model trained in one environment less accurate when applied to another. When training a model, a polytunnel’s historical data may offer valuable insights, but it may not be relevant if the configurations are different. For example, in one of our sensor deployments, the polytunnel had grown a different plant variety years before with a very different growth cycle, and the historical data would thus not be relevant for use. Similarly, we have previously demonstrated the need for effective domain adaptation approaches where AI models are used to monitor fermentation process in craft breweries with varying formulations [20].

## 6. Data Quality

The importance of good data in AI cannot be overstated. Accurate forecasts rely on comprehensive, up-to-date, and relevant data. With machine learning models, acceptable error margins for predicting strawberry yield can be difficult to accomplish, as the quality of real-world data are often profoundly affected by incomplete fields, misclassification, and systematic omissions [21]. Data sparsity is also an issue, as although soft-fruit growers are consistent in usually harvesting twice a week, this would only yield, at best, 20 data points per tunnel for a good season. Data limitation issues here can be compounded when the dataset is limited to a specific variety of strawberries. This reinforces how important investing in the data collection process is, as research has shown that decreases in data quality, such as completeness, feature accuracy, and target accuracy, can significantly impair algorithm performance, especially within regression problems [22].

While the sensors we deployed generally captured continuous data at set intervals, some sensors had missing data due to a variety of technical issues. For instance, one of the reliability issues we faced with the deployment of our light sensors was connectivity and the consistency of readings, as unlike the other sensors we deployed, the light sensors were each powered by a small individual solar panel. As the plants grew around the sensors, they created more shadow and affected the light levels available to the solar panels that were powering them. This would then lead to issues where some of the light sensors now would not receive enough power and shut down either completely or for extended periods, depending on how covered the solar panel was. Another consistency issue we had with the sensors was that although all of the light sensors were set up the same, a small selection of the sensors would sporadically give noisy and incorrect readings, which were believed to be due to a hardware issue. The soil moisture sensors also provided a challenge, often needing attention and recalibration due to the unique characteristics of different soil-related problems. These problems ranged from air pocket formation to variability in soil composition, all of which can significantly impact the accuracy of the collected data. In any future work, the soil moisture data would have to be carefully monitored and engineered during data processing, and a correction factor may be applied if an offset is present. In the presence of an offset, manual data engineering becomes necessary. For instance, the average soil moisture may be computed if the sensors have very different values but similar trends. However, if the trends are different, even more complex troubleshooting would be required, such as determining if one area is receiving more water and then engineering accordingly. This type of data engineering cannot be automated, as it often requires critical analysis and each case may be treated differently.

Such external factors affecting sensor deployments in the agri-food context can hinder the data collection process, leading to missing data, suboptimal machine learning models, and negative impacts on the scaleability of the AI solution.

## 7. Discussion and Future Perspectives

### 7.1. Barriers and Challenges to Adoption of Novel Technological Approaches

As mentioned before, developing new technological solutions cannot happen in a vacuum. Proper user engagement throughout the process is crucial for eventual real-life deployment and user acceptability. Growers and farmers need to be part of the co-creation process and they require a clearly defined value proposition in order to implement, deploy, and maintain new technologies. Disrupting and changing their ways of working is sometimes a long process that is dependent not only on model performance, but also on complexity, usability, and reliability. New methods, such as those including AI, depend on high-quality data, as the final product will represent the quality of data that were used for training.

#### 7.1.1. Automation in Carbon Footprint Monitoring

The measuring/monitoring, reporting, and verification (MRV) of emissions represents an additional significant burden to the agri-food sector in addition to long-term staff shortages, food price volatility, and changing climate. Automating the collection, analysis, and reporting of sustainability data may relieve much of this burden; however, this is not yet possible at scale across the agri-food sector. Barriers preventing such automation in practice that we have observed in our case studies include the following:

- **Capital costs** required to improve the monitoring capabilities of businesses. While the current focus is mainly on software-based solutions (e.g., carbon footprint calculators), these currently require large volumes of manually entered data (e.g., field observations). Therefore, to achieve full automation, it is likely that significant investments in hardware (e.g., smart meters to monitor individual pieces of heavy machinery) will be required.
- Wide **variety** in the “controllability” of the food production environment. For example, while breweries are a good example of well-contained nearly “lab-like” production environments where all processes can be closely monitored and controlled, strawberry production takes place in a technology-free “unconnected” setting (i.e., polytunnels scattered across large area).
- A lack of **standards** and **regulations** that would set clear future-proof expectations of the type of monitoring that needs to be in place.
- The **effectiveness of sensor technologies** in sustainability and resource monitoring still faces significant challenges, especially in more demanding environments. For example, energy monitors are increasingly utilised within residential settings, yet adapting these systems to commercial or industrial contexts, where devices may need to be positioned outdoors or transmit data over extensive distances, remains a challenge.

#### 7.1.2. AI-Based Decision Support

AI models are especially suited to decision-making support; however, their real-world application is still lacking in most cases. The barriers that we have observed in our case studies include the following:

- **Model generalisation** is one of the primary challenges in deploying AI across diverse agricultural settings due to the variability in agricultural practices across the industry, leading to data sets with different volumes, varieties, and complexities [23,24]. These varied and inconsistent data can affect the model’s generalisation capabilities [25]. A global model trained on data collected from a broad spectrum of farms will find common patterns and correlations that apply to all these environments. However, this can lead to underfitting when such a model is applied to specific farms with unique characteristics not adequately represented in the dataset, meaning the model has worse performance on these farms. Alternatively, models fine-tuned for optimal performance on a single farm may overfit, resulting in poor performance when applied to different farms with varied soil types, polytunnel settings, and soft-fruit varieties. This dilemma not only hinders the effectiveness of AI solutions, but also limits their scalability across the agri-food sector.
- **Economic viability**: The costs from collecting and processing data to analysing and developing AI solutions can be prohibitive, particularly for small to medium-sized enterprises, which constitute a significant portion of the agri-food industry in the UK. Furthermore, the models may progressively perform worse due to changes in environmental factors. The ongoing maintenance and necessary iterative improvement of these models add additional layers of cost and complexity. For agri-food growers to consider employing such AI solutions, they must be economically viable and widely adopted. They must not only demonstrate clear benefits in terms of yield improvement and resource optimisation but also be cost-effective and accessible.
- **Domain-specific knowledge** from agronomists and experienced farmers can significantly improve the accuracy and utility of models. This helps tailor the models to reflect the realities of individual farms. For instance, expert input can guide the selection of features that are most predictive of yield in strawberry production or critical for process optimisation in brewery operations. Farmers from individual farms are often responsible for manually creating and updating their yield predictions. These forecasts and the associated updates contain a lot of extra latent information, and using these forecasts as an input may further enhance the accuracy of models.
- **Ethical and practical concerns** are raised when deploying AI in agriculture. AI models require a large quantity of data, collected across many farms. The sharing of such data raises concerns regarding ownership and control. Farmers might be hesitant to share their data, fearing misuse or exploitation. One way to address this is via the use of federated learning, where individual models are trained locally and aggregated globally [26]. While overall performance is improved with the use of more data, data privacy is also preserved, since the data never physically leave the farms. There is also a risk of over-reliance on technology, where blindly following AI recommendations without human oversight can result in undesirable outcomes. AI models, while useful, are not infallible. For instance, they may not account for unexpected events such as heat waves or other environmental anomalies leading to unusual outcomes. AI should be seen as a tool to guide decision-making, enhancing rather than substituting the expertise and judgement of farm staff.

#### 7.1.3. Social Factors

Although technical challenges are still substantial, the broader and often overlooked social challenges in the agri-food sector are common, which is deeply affected by diverse factors such as high heterogeneity, public policies, local traditions, and an unstable economic landscape. The key observed challenges include the following:

- A **lack of enthusiasm or drive** among businesses to invest in sensors and AI technologies for improving sustainability. This largely stems from the need to prioritise profit and economic viability above environmental concerns. Most companies will be hesitant to allocate resources to sustainability measures unless they can see a direct link to financial benefits or regulatory compliance. Therefore many companies are unaware of possible sensing solutions, and the most developed sensing technologies are usually those that monitor product quality and therefore have a more direct impact on the economics of the business.
- **Unawareness** of available AI and sensor technologies and their benefits. Many businesses in the agri-food sector are not fully informed about the types of sensors available (e.g., fermentation sensors, electricity monitors, and water meters) and the advantages they offer (e.g., direct inputs into carbon calculators to identify emission hotspots, economic benefits through reduced resource usage, and enhanced equipment scheduling efficiency). Where decision support systems used by farmers do exist, these often do not embed any advanced AI methods, but simply display the collected sensor data.
- The **knowledge gap** between researchers working on sensors and AI and business operators who lack awareness of the potential of such technologies. This gap hinders effective communication and collaboration between researchers and industry practitioners, leading to missed opportunities for innovation and improvement. This makes it difficult to identify the most promising areas for sensor application or to envisage the potential benefits that digital technologies could bring to the business.
- **Ownership** of the tools (e.g., carbon footprint calculators, AI models) might be unclear. While the tools for managing the impact of GHG emissions should be freely available to ensure widespread adoption, the data used for their development are costly. Farmers or developers might be looking for compensation in terms of licence fees, which, in turn, may hinder the transparency of, for example, the emission calculation methods and data used to produce the emissions estimates.

### 7.2. Future Directions

Below, we briefly outline some of the promising AI-based applications that we are exploring further in the agri-food context.

#### 7.2.1. Semantic Data Integration and Transparent Carbon Footprint Calculations

The challenge related to the scarcity of good-quality data coupled with the culture of data silos in the agri-food sector could be addressed by implementing an interoperable “smart data layer” based on semantic web technologies. Such an approach would require the development and adoption of new farm management support tools that would describe the existing local data using standard vocabularies to produce knowledge graphs [27]. This would enable an unambiguous digital representation of the agri-food sector with opportunities for the seamless tracking of different types of provenance information about business operations and product lifecycles. A number of existing ontologies could be reused by the potential solutions; for example, the Agriculture Information Model (AIM) [28] provides a vocabulary to describe agricultural assets (e.g., farm, parcel) and their properties (e.g., types of plants grown). Similarly, existing standards for describing sensors and their observations, such as W3C Semantic Sensor Ontology (SSN) [29] and W3C PROV [30] for describing causal provenance graphs, can be extended to provide agri-food-specific data representation models.

Our initial work in this direction focuses on capturing detailed descriptions of the emission calculation processes by leveraging the SSN and PROV vocabularies [15]. Semantic representations could be also further exploited to support automated transparent carbon footprint estimates of food products and agri-food operations. For example, in [16] we demonstrated how semantic descriptions of emission conversion factor ontology and a farm asset (e.g., irrigation rig) including its static properties (e.g., asset type, power rating, deployment location, etc.), as well as descriptions of dynamic observations over time (e.g., amount of electricity consumed by a production line), could be automatically mapped as inputs of semantic emissions calculation formulas. Such semantic descriptions of various data inputs and formulas guiding calculation processes then enable the automated creation of transparent carbon footprint estimates associated with a detailed data provenance trace.

#### 7.2.2. Synthetic Data Generation for Training AI Models

The volume of the training set is crucial for the performance and generalisation power of AI models. Most researchers and farmers have limited sensor data, which makes it difficult to train robust models. When the data volume is insufficient, it is critical to investigate a solution to enhance the dataset. The generation of synthetic data could be leveraged to improve the accuracy of the forecasting models. Techniques such as data augmentation [31] and backcasting [32,33] are widely used to create artificial data. Data augmentation is a technique that slightly modifies the original data by applying various transformations while retaining their labels or annotations. For example, Generative Adversarial Network-based augmentation has been previously applied to increase training samples to improve winter wheat yield forecasting [34]. In data backcasting, an AI model is employed to predict missing historical feature values. For example, consider an ML yield-forecasting model trained on data inputs observed by IoT sensors such as soil moisture, the polytunnel’s internal humidity and temperature, etc.; however, the sensor data are available only for one season. We could apply backcasting techniques to generate missing historical sensor values for previous seasons by utilising available proxy measurements such as the local historical weather station data, which are typically available from national weather services. These new backcasted data could then be used to enhance the training set containing the original sensor data.

#### 7.2.3. Multi-Objective Optimisation for Carbon-Aware Decision Support

Agri-food producers are increasingly tasked with conflicting objectives. On one hand, they want to maximise their yields; on the other hand, they want to reduce their carbon footprint. In this context, multi-objective optimisation (MOO) offers an approach to managing trade-offs between yield and associated carbon costs such as water, electricity, fertiliser usage, etc. Such a system would utilise algorithms to identify Pareto-optimal solutions, which represent scenarios where no objective can be improved without worsening another. For agri-food growers, this means identifying a set of practices that achieve the best possible yield without disproportionally increasing carbon costs.

Consider a strawberry farm whose objective is to maximise yield while minimising the carbon footprint associated with water and fertiliser usage in the polytunnels. By applying MOO, farm management can evaluate different cultivation strategies and fertiliser applications to find the optimal balance. For instance, if the model suggests that decreasing water usage by 10% only decreases yield by 1%, this may be an attractive option, as it significantly reduces carbon costs. Such insights can assist farmers in making informed decisions that align with their productivity targets and sustainability commitments.

Algorithms such as NSGA-II (Non-dominated Sorting Genetic Algorithm II) or MOEA/D (Multi-Objective Evolutionary Algorithm based on Decomposition) [35,36] need to be explored for their efficiency and explainability to evaluate multi-dimensional trade-offs in agri-food settings. In our experimental pipeline, we simulate yield output and water usage associated with environmental factors, such as those from our sensors, with machine learning models. The MOO algorithm then evaluates these scenarios and plots the Pareto front, allowing us to visualise the trade-offs between maximising yield and minimising carbon costs. With sufficient data collected in the future, we will build the aforementioned system and evaluate this pipeline with real data.

### 7.3. Call to Action

Technical issues related to model generalisation, and the quality and quantity of data required for AI systems, are frequently listed as limitations of approaches combining AI and IoT in the agri-food context [37,38]. Some of the wider socio-technical challenges we have experienced, such as a lack of expert skills, the costs of new technologies, and connectivity issues, were also highlighted [38], and these are consistent with the discussions of barriers to wider IoT deployments in agri-food [39,40]. In our study, we expand this discussion with additional challenges, such as those related to lacking regulation, a lack of integration of domain-specific expert knowledge, unawareness, etc.

However, overcoming many of these challenges will likely require a significant amount of time (e.g., collecting data over several years), and addressing each challenge in isolation is not feasible. Initiatives such as the 2050 net zero targets impose challenging time constraints on these technologies should they play a significant part in our emission reduction efforts. We theretofore call for urgent integrated interdisciplinary efforts to enhance the adoption of AI in agri-food to increase the sustainability of the sector. In this context, we deem our holistic socio-technical view—with novel perspectives on, for example, data granularity, the transparency of emission calculation processes, and the role of synthetic data generation and semantic technologies—to be an important contribution to bridging the gap between the requirements of AI models, data availability, and the existing emission calculations.

## 8. Conclusions

Our perspective discussed in this paper is based on specific agri-food domains that produce non-staple items, which may hinder their perceived importance. However, the soft-fruit and beer industries are important contributors to the UK economy, and this part of the agri-food sector must not be left behind in our race towards net zero. We could also argue that the various data quality-related issues mentioned in the strawberry use case could be solved by a “big data” approach where we simply scale up sensor deployment and generate more data. However, although our experimental deployments were limited to only a few polytunnels, this kind of scenario is typical of what a farmer would do in practice. It is unfeasible to expect that hundreds of polytunnels would be equipped with expensive and potentially unreliable technology. This in itself would represent not only significant capital costs, but also a secondary carbon footprint and increased maintenance costs over time. Instead, smaller deployments where the results can be generalised to the whole field are perhaps more realistic.

We have also predominately focused on our experience of building IoT-based AI decision support systems gained within the UK context. However, the monitoring requirements for carbon footprint reporting are often set in accordance with international standards (e.g., IPCC methods), and hence, many of the challenges discussed in this article will also apply to a wider international context. The challenges linked to economic viability and social factors preventing AI deployments may become even more pressing in less developed nations due to the lower availability of resources and skills to implement net zero technologies [41]. Finally, our prototype solutions focused on data-centric technologies for the AI-based decision support of human operators. IoT sensors were restricted to inexpensive and battery-operated devices that can be deployed in a remote location (e.g., a polytunnel) without a mains electricity supply, as we believe that this kind of setting covers a large portion of agri-food operation in the UK. Technologies such as the image-based monitoring of plants’ cycles using cameras, farm robots, drones, and other hardware-heavy solutions were considered out of the scope of our project.

To conclude, AI technology is not a panacea for net zero challenges in agri-food. However, if used correctly, it has the potential to become a useful tool among other process-based modelling approaches and existing technical solutions, such as sensing technologies, which can benefit from AI’s ability to automate, explain, and scale complex computations. Affordable automation at scale is key to the wide-scale adoption of net zero decision support systems.

## Data Availability

No analysis is presented in this paper.
